# Effects of 4-Week Inspiratory Muscle Training on Sport Performance in College 800-Meter Track Runners

**DOI:** 10.3390/medicina57010072

**Published:** 2021-01-15

**Authors:** Yun-Chi Chang, Hsiao-Yun Chang, Chien-Chang Ho, Po-Fu Lee, Yi-Chen Chou, Mei-Wun Tsai, Li-Wei Chou

**Affiliations:** 1 Department of Physical Therapy and Assistive Technology, National Yang-Ming University, Taipei 112, Taiwan; james76630@gmail.com (Y.-C.C.); tmwk@ym.edu.tw (M.-W.T.); 2 Department of Physical Education, Fu Jen Catholic University, New Taipei City 242, Taiwan; 093703@mail.fju.edu.tw (C.-C.H.); f520184fred@yahoo.com.tw (P.-F.L.); 3 Department of Athletic Training and Health, National Taiwan Sport University, Taoyuan 333, Taiwan; yun1130@ntsu.edu.tw; 4 Research and Development Center for Physical Education, Health, and Information Technology, Fu Jen Catholic University, New Taipei City 242, Taiwan; 5 Graduate Institute of Sport Coaching Science, Chinese Culture University, Taipei 111, Taiwan; 6 Physical Education Office, National Tsing Hua University, Hsinchu City 300, Taiwan; a0925701007@yahoo.com.tw

**Keywords:** respiratory muscle capability, athletic performance, muscle fatigue

## Abstract

*Background and objectives:* Respiratory muscle fatigue is one of the important factors limiting sports performance due to the metaboreflex. This reflex will cause a decrease in blood flow to the extremities and accelerate exercising limb fatigue. Previous studies found that inspiratory muscle training (IMT) can effectively enhance the respiratory muscle endurance and reduce fatigue during long-duration exercise or aerobic exercise, thereby enhancing athletic performance. However, the mechanism between inspiratory muscle strength, change of limb blood flow and sports performance still requires investigation, especially in short-duration exercise, anaerobic or both aerobic and anaerobic exercise. The purpose of this study was to investigate the effects of 4-week inspiratory muscle training on respiratory muscle strength, limb blood flow change rate and sports performance in recreational 800-m college runners. *Materials and Methods:* Twenty healthy 800-m college runners randomized into the IMT group (11 subjects) and control group (9 subjects). IMT consisted of 30 inspiratory efforts twice daily, 5 days a week, with intensity at 50%, 60%, 70% and 80% of maximum inspiratory pressure (MIP) for 4 weeks, while a control group kept 50% of MIP for 4 weeks. An 800-m trial test, limb blood flow change rate by using Impedance Plethysmography, and MIP were as the outcome measured variables and be evaluated. All measured variables were assessed before and after 4-week IMT training. Two-way ANOVA was conducted for statistical analysis. *Results:* The results showed significantly interaction between groups and pre-posttest. IMT group significantly decreased limb blood flow change rate from 19.91 ± 11.65% to 9.63 ± 7.62% after received the IMT training program (*p* < 0.05). The MIP significantly improved from 112.95 ± 27.13 cmH_2_O to 131.09 ± 28.20 cm H_2_O in IMT group, and the 800-m trial test also shorted the running time from 162.97 ± 24.96 s to 156.75 ± 20.73 s. But the control group no significantly changed in MIP and 800-m trial test. *Conclusions:* Our results indicated that the 4-week IMT training (twice a day, 5 days a week) significantly improves participants’ inspiratory muscle strength, 800-m running performance and decreases the limb blood flow change rate.

## 1. Introduction

Jogging has become one of the popular activities and aerobic exercise in the worldwide recently. As an aerobic-based exercise, cardiopulmonary endurance and respiratory muscle strength are fundamentally required and should develop to enhance cardiorespiratory health. The diaphragm, one of the respiratory muscles, is essential to and functions as the skeletal muscles for respiratory movement [1,2]. While individuals engage in high-intensity exercise or experience a period of effort, the fatigue of respiratory muscles will reflexly increase sympathetic vasoconstrictor activity and vasoconstriction of the vasculature of the exercising limb, as a result, blood flow cannot reach the muscles of the limbs [3,4,5,6]. Therefore, insufficient blood flow in limbs decreases the oxygen exchange rate and increases the feelings of soreness and discomfort, thereby affecting their sports performance.

According previous studies, during constant-load exercise, the work of respiratory muscle was reduced about 60% by proportional assisting ventilation, and end-exercise quadriceps fatigue decreased by 25–30% compared with the control group [7]. Furthermore, during constant-load leg cycling (adding supplemental O2 to the inspired air), when exercise-induced arterial desaturation was prevented, it was found that quadriceps fatigue was nearly 50% less compared with the control group [8]. Thus, training for the respiratory muscles may extend the period of fatigue, thereby improving sports performance, balance of arterial blood, gas and acid–base [9].

From the athletic perspective, previous studies have indicated that inspiratory muscle training (IMT) significantly reduces fatigue and improves athletic performance. Johnson and colleagues [10] used the threshold IMT (PowerBreathe^®®^) (POWERbreathe International Ltd., England, UK) intervention for 6 weeks on cyclists. Their results indicated that after six weeks of training, the endurance the continuous power output increased and cycling performance improved. The same results were found in Romer and colleagues’ work [11]. In this study, sixteen trained cyclists were randomly assigned into IMT group and sham IMP group and received 6-week IMT training. The pulmonary function and 20 and 40 km time-trial performance were measured. The result improved the 20 and 40 km time-trial performance and pulmonary function. Kilding et al. also investigated the IMT effect on 16 competitive club-level swimmers. Their results also are similar those previous studies. Their results indicated that decreased the timing in the 100 m swim by 1.7% and in 200 m swim by 1.5% [12]. In addition, Volianitis and colleagues [13] conducted an 11-week of threshold IMT (PowerBreathe^®®^) on elite female rowers. Their results of the maximum inspiratory pressure in the IMT group were significantly higher than in the control group. It can be seen that IMT can improve respiratory muscle strength and sports performance.

In past studies, the exercises for respiratory muscle training included running, swimming, and cycling [14,15,16]. Most of them focused on long-distance and aerobic exercise. However, there were fewer studies on respiratory muscle training for middle-distance and short-distance sports. Middle-distance running events include 800 m and 1500 m. The 800-m and 1500 m distance are the sport that includes both aerobic and anaerobic exercise event. A study for 1500 m runners has proved that respiratory muscle training can increase respiratory muscle strength and improve athletic performance [15]. However, the distance of 800 m is shorter than the distance of 1500 m. In terms of exercise physiology, it tends to more anaerobic exercise [1,2,14]. It is doubtful whether the effect of respiratory muscle training is as same as 1500 m distance.

However, previous studies related IMT effect have focus on long-duration exercise. Few studies investigate the effect of IMT on shorter distance sports, like 800-m distance run. Brown and Kilding found that the occurrence of inspiratory muscle fatigue after a short-duration front crawl swim exercise [14]. Ohya et al. also reported that shorter-duration running exercise would induce inspiratory muscle fatigue [17]. Therefore, there is a need to better understand whether respiratory muscle training is helpful for middle distance events, like 800-m distance run. Further investigation is still needed to stablish the possible mechanism. Hence, the purpose of the study was to investigate the effects of 4-week inspiratory muscle training on respiratory muscle strength, limb blood flow change rate and sports performance in recreational college 800-m track runners. The research hypothesis was 4-week inspiratory muscle training increased respiratory muscle strength, decreased the limb blood flow change rate and improve 800-m sport performance.

## 2. Materials and Methods

### 2.1. Study Design and Participants

This study was conducted with a two group compared, pre and post- test design. According to the basic statistical power requirement from a previous study [18], the minimum number of subjects in each group should be at least 8. Finally, twenty-two recreational 800-m college runners from the track and field Team of the university were recruited. After randomization of the IMT group and control group, two participants dropped out of the control group. Participants were recruited by finding athletes who had trained at least three times per week for a minimum of 60 min and were able to finish the full experiment. Moreover, three exclusive criteria were applied: (1) present or past cardiovascular, pulmonary and neurological disease; (2) smoker; (3) allergies to electro-conductive pads. All participants were informed of the study procedure and signed the informed consent form before the experiment. The present study was also approved by the Institutional Review Board of China Medical University Hospital in Taiwan (CRREC-102-027, 17 June, 2014). Table 1 presents the anthropological characteristics of the participants. No significant difference was found between the IMT group and the control group.

### 2.2. Procedure

**Figure 1:**
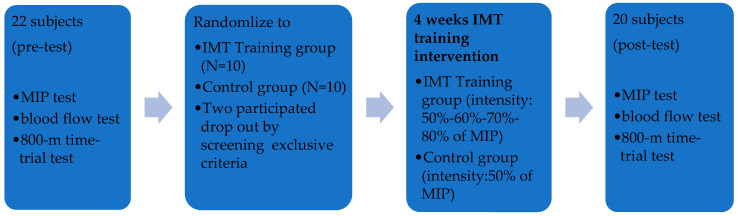
Experimental Procedure.

Baseline data of the inspiratory muscle training (MIP) test, blood flow test and 800 m time-trial test were collected from all participants before inspiratory muscle training intervention. After Baseline data collection, all participants take a day’s rest, the training group started a 4-week inspiratory muscle training and the control group started placebo training. Both groups did the same skill training and weight training for 4 weeks. All participants repeated data collection from MIP test, blood flow test, and 800 m time-trial test immediately after 4-week IMT. The procedure flow list in Figure 1.

### 2.3. Measurements

#### 2.3.1. Maximal Inspiratory Pressure (MIP) Assessment

The MIP assessment in the present study was measured by the MicroRPM (Micro Medical/CareFusion, Kent, United Kingdom) with inserted the PUMA PC Software (Micro Medical, Kent, England) in the laptop. The PUMA PC software works with the MicroRPM Respiratory Pressure Meter to measure respiratory muscle strength and calculated the MIP from the one-second average maximum pressure. During the test, the participants should be sitting, then the investigator explained the MicroRPM device usage procedure to the participants. When performed the MIP assessments, participants were asked to exhale slowly and completely first with sealed lips around the mouthpiece, and then inhale hard rapidly. The investigators record the data for the largest value which show on the MicroRPM and its software. Participants were allowed to rest between each trial for 1 min and were asked to repeat the protocol 5 times [19]. Accord to the previous study, the MicroRPM reliably measured MIP [20].

#### 2.3.2. Limb Blood Flow Assessment

**Figure 2:**
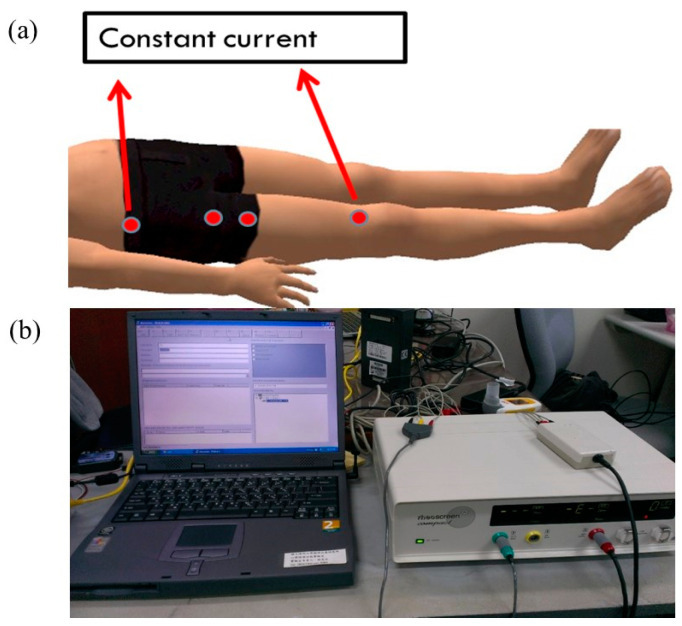
The location of electrodes (**a**) and equipment (**b**) during the limb blood flow measurement.

Impedance Plethysmography RheoScreen compact (Medis, Ilmenau, Germany) was used to evaluate limb blood flow. The device had been calibrated and guaranteed high quality of the recorded signal, as well as high reproducibility [21]. The test used measurement by a four-electrode technique. The two outer electrodes were used to apply the current, and the two inner electrodes were used for delineating the quadriceps under measurement (Figure 2).

To clearly assess limb blood flow change rate among the participants, every single assessment was conducted 2 times and cooperated with the respiratory muscle fatigue induction. After the first blood flow assessment, the participants were asked to breathe in, maintaining 60% of inspiration pressure by the POWERbreathe respiratory training device. The intensity of the inspiration pressure was based on the MIP assessment, and the time ratio of inspiration and expiration was 1:2. Participants were encouraged to maintain their breath. Once the participant failed to maintain the inspiration pressure more than 2 times, the process was ended. Then, the lower limbs’ blood flow was immediately measured. The limb blood flow change rate were calculated the value of the second test minus the value of the first test, then divided by the value of the first test, and multiplied by 100%.

#### 2.3.3. Athletic Performance Assessment

An 800-m time trial test was used as the athletic performance assessment and was carried out before and after four-week respiratory muscle training. According to studies from Hanon et al. and Ohya et al., the 800-m track running created a state of imbalance within the body, a decline in blood pH and the excessive functioning of certain compartments, which leads the body to exhaustion and respiratory muscle fatigue [17,22]. Therefore, this athletic performance test can be used as an outcome measured variable of the effectiveness of respiratory muscle training. Before the 800-m time trial test, the participates warmed up for 5 min and then performed the test on the athletic track on campus. During the test, they ran 800 m on the track and recorded the time with a timer.

### 2.4. Interventions of Respiratory Muscle Training

This study adopted a resistance-adjustable electronic respiratory training device called POWERbreathe K2 (POWERbreathe International Ltd., England, UK) for the training intervention. Initially, the participants were asked to hold the disposable air nozzle with their mouth and applied a nose clip to prevent ventilation through the nasal passage. When the primary setting was done, the participants were allowed to start breathing following the signals from the device and the investigators. The device provided resistance during inhalation while recording inspirational pressure. Participants were allowed to discontinue instantly once they felt any discomfort.

This intervention was completed twice a day, 5 days a week for the IMT group. The training intensity was progressively set at 50%, 60%, 70% and 80% of MIP for weeks 1, 2, 3 and 4, respectively. Further, the control group was required to keep at the level of 50% MIP for 4 weeks.

### 2.5. Statistical Analyses

The Statistical Package for Social Sciences (SPSS 20, SPSS Inc., Chicago, IL, USA) software was used for the statistical analysis. The participants’ anthropological information was first described, with the independent sample t-test and chi-square test in order to compare the group differences. The normality of variables was evaluated with the Shapiro–Wilk test. Subsequently, two-way ANOVA was conducted for the pre- and post-test results and group comparison. Data are presented as mean and standard deviation for continuous variables and as ratios for nominal variables. A significance level of α = 0.05 was adopted

## 3. Results

MIP, blood flow change rate and 800 m data were expressed as mean and standard deviation (Table 2). Table 3 shows the results of respiratory muscle training in the present study. After a 4-week respiratory muscle training, all variables were found significantly interaction effect between group and pre-post training (*p* < 0.05). This indicated that the MIP significantly improved after 4-week respiratory muscle training.

## 4. Discussion

The purpose of this study was to investigate the effects of 4-week inspiratory muscle training between respiratory muscle strength, limb blood flow change rate and sports performance in recreational 800-m college runners. Our results indicated that 4-week IMT significantly increase MIP and improve 800-m run performance and decrease the limb blood flow change rate. The present study explained and completed the phenomenon which has seldom been discussed before.

According to our results, the IMT group significantly improved MIP from 112.95 ± 27.13 cm H_2_O to 131.09 ± 28.20 cm H_2_O, but the control group did not. One meta-analysis study has previously pointed out that respiratory muscle training enhances MIP particularly for endurance exercise [23]. The 800-m distance is a sport that includes both aerobic and anaerobic exercise event [14]. Based on our study results, 4-weeks respiratory muscle training enhanced the sports performance of the 800 m middle-distance running. This result had not appeared in previous studies.

Previously, a systematic review suggested that 4 to 12 weeks may be a proper duration for respiratory muscle training [23]. In our study, only 4 weeks of IMT with setting intensity at 50%, 60%, 70% and 80% of MIP, we were able to observe significant improvements. These results were consistent with previous studies. The previous research group comprised elite athletes who could quickly learn breathing muscle training skills, so it only needed 50% intensity and 4 weeks of training time to see progress. For the other studies, it took 6–8 weeks for recreational runners. The training effect can be achieved when the intensity was above 80% [1,15]. Specifically, a previous study set 50% of MIP as the intensity for the training group, resulting in significant improvement [24]. However, in our study, the control group was also recreational runners, and set at 50% of the training intensity for 4 weeks was not enough to see significant results. It was different with previous studies. It may indicate that recreational runner needs more time and training intensity to learn breathing training skill. The possible mechanism is not clear. The future study needs to clarify the effect of training skill on respiratory muscle between recreational and professional runners.

On the other hand, the limbs’ blood flow significantly decreased from 19.91 ± 11.65% to 9.63 ± 7.62% after the IMT training program. Conversely, the control group increased from 5.33 ± 7.45% to 13.50 ± 7.48%. It may indicate that consistent intensity does not improve respiratory muscle function and decrease blood flow change rate in control group. In general, a maximal exercise may result in vasoconstriction in locomotor muscles [25] and cause peripheral fatigue and, in part due to accompanying high levels of respiratory muscle working, thereby increases the blood flows [8]. Therefore, we found that the control group at a consistent respiratory muscle training intensity did not affect respiratory muscle endurance, which in turn affected the amount of blood flow change rate. However, after the 4 weeks of IMT training, the blood flow changing of the IMT group were reduced. The possible reason is that the running movement is an upright dynamic pattern on land. It was required core muscles involved during running, including the diaphragm (both respiratory muscles and trunk core muscles) [17]. With IMT training, it progresses from 50% to 80% intensity, it can increase the vertical movement of the diaphragm during this resisted training for respiratory muscles and may promote the perfusion of blood flow to the limbs, which reduce the influence of metabaroreflex phenomenon. This implies that the training of incremental IMT intensity attenuated metaboreflex phenomenon and reduced lower limb blood flow change rate in recreational runners.

The significant improvement in the 800-m running performance from 162.97 ± 24.96 sec to 156.75 ± 20.73 s was only observed in the IMT group. The same result has been previously observed. In particular, Nicks and colleagues performed 5-week respiratory muscle training in soccer players and examined their fitness performance by Yo-Yo Intermittent Recovery Test. The performance was significantly improved in their study [26]. Similar results have been found in another approach with 4 weeks and 6 weeks of respiratory muscle training [27,28]. A meta-analysis has also demonstrated a significant positive effect of IMT for fitness performances [23]. Generally, our results are consistent with previous research. To conclude, 4-weeks IMT training (twice a day, 5 days a week), progressively from 50% to 80% of MIP improved 800-m performance in college recreational runners.

The strength of the present study is the participants and sports performance variable selection. We chose the 800-m timed trial test and 800-m recreational runners. This track event has belong to both aerobic and anaerobic exercise types. Our results also proved that the respiratory muscle training is not only effective for endurance exercises, but also for mixed forms of exercise (both aerobic and anaerobic exercise types). However, some limitations should be addressed. First, the present study recruited runners with only one single race in a particular range of age. Future studies are encouraged to investigate different races, ages, cultures, and types of athletes in order to ensure the cross-validation of the mechanism. Second, although we have minimized possible interferences in the study, the consistency of individual factors such as dietary and sleep duration could not be guaranteed. It may interfere with the results of the research.

## 5. Conclusions

The present study conducted a 4-week respiratory muscle training intervention in order to investigate its effects on sports performance for recreational runners. Our results indicated that the 4-week IMT training (twice a day, 5 days a week) significantly improves participants’ inspiratory muscle strength, 800-m running performance and decreases the limb blood flow change rate. A possible mechanism that increasing MIP could delay the onset of respiratory muscle fatigue and help reduce lower limb blood flow change rate, finally improved 800-m sport performance.

## Figures and Tables

**Table 1:** The detailed information of the participants (*N* = 20).

Anthropological Information	IMT Group (*N* = 11) Mean ± SD	Control Group (*N* = 9) Mean ± SD	*p*-Value
Gender (M:F)	8:3	6:3	0.10
Age, years	21.64 ± 2.06	20.78 ± 1.48	0.31
Height, cm	170.59 ± 6.7	172.33 ± 9.94	0.64
Weight, kg	61.46 ± 6.92	63.39 ± 14.33	0.69

Abbreviations: SD, standard deviation.

**Table 2:** The results of the MIP, blood flow change rate, and 800-m times trial test between IMT and control group.

	IMT * Group		Control Group	
	Pre (*N* = 10) (Mean ± SD)	Post (*N* = 10) (Mean ± SD)	Pre (*N* = 10) (Mean ± SD)	Post (*N* = 10) (Mean ± SD)
MIP (cmH_2_O)	112.95 ± 27.13 ^†^	131.09 ± 28.20 ^†^	116.33 ± 40.56	117.00 ± 36.40
Blood flow change rate (%)	19.91 ± 11.65	9.63 ± 7.62	5.33 ± 7.45	13.50 ± 7.48
800-m test (sec)	162.97 ± 24.96	156.75 ± 20.73	166.67 ± 21.83	167.60 ± 20.73

^†^: there is significant difference between pre-training and post-training in IMT groups. * IMT: Inspiratory Muscle Training.

**Table 3:** The repeated measure two- way ANOVA results of the MIP, blood flow change rate, and 800-m times trial test between IMT and control group.

	Within-Subject (Pre-Post)			Between-Subject (Group)			Interaction (Group × Pre-Post)			
	F	*p* Value	Partial Eta Squared	F	*p* Value	Partial Eta Squared	F	*p* Value	Partial Eta Squared	Power
MIP (cmH_2_O)	10.966	0.004 *	0.379	0.136	0.717	0.007	9.466	0.007 *	0.345	0.829
Blood flow change rate (%)	0.272	0.609	0.015	2.443	0.135	0.119	20.691	0.000 *	0.535	0.990
800-m test (s)	3.932	0.063	0.179	0.541	0.471	0.029	7.174	0.015 *	0.285	0.717

* *p* < 0.05.

## Data Availability

The data that support the findings of this study are available on request from the corresponding author. The data are not publicly available due to privacy or ethical restrictions
